# Resilience and Perceived Social Support in Cancer Survivors: Validity, Levels, and Sociodemographic Correlates of CD-RISC-25 and MSPSS Scales

**DOI:** 10.3390/healthcare13212698

**Published:** 2025-10-25

**Authors:** Goran Malenković, Jelena Malenković, Sanja D Tomić, Silvija Lučić, Armin Šljivo, Fatima Gavrankapetanović-Smailbegović, Slobodan Tomić

**Affiliations:** 1 Faculty of Medicine, University of Novi Sad, 21000 Novi Sad, Serbia; goran.malenkovic@mf.uns.ac.rs (G.M.); jelena.trivkovic@gmail.com (J.M.); sanja.tomic@mf.uns.ac.rs (S.D.T.); silvija.lucic@mf.uns.ac.rs (S.L.); 2 Clinical Center of University of Sarajevo, 71000 Sarajevo, Bosnia and Herzegovina; sljivo95@windowslive.com (A.Š.); fatima.smailbegovic@gmail.com (F.G.-S.)

**Keywords:** breast neoplasms, resilience, social support, survivors, patient-reported outcomes measures

## Abstract

**Background and Objectives**: Resilience and perceived social support are crucial factors influencing psychological well-being among breast cancer survivors. Understanding their levels and interrelations can inform psychosocial interventions aimed at improving survivorship outcomes. This study aimed to examine the relationship between resilience and perceived social support, to evaluate the psychometric properties of the applied scales, and to explore their associations with key sociodemographic factors among breast cancer survivors. **Materials and Methods**: A total of 193 women in clinical remission, at least six months post-primary treatment, were recruited from the General Hospital Sombor. Participants completed sociodemographic and clinical questionnaires, the Connor–Davidson Resilience Scale (CD-RISC-25), and the Multidimensional Scale of Perceived Social Support (MSPSS). Descriptive statistics, Pearson’s correlations, and group comparisons (*t*-tests and ANOVA) were conducted to assess the relationships among study variables and sociodemographic factors. **Results**: Participants demonstrated moderate resilience (57 ± 18), with Coping and Hardiness as the strongest domains and Optimism the lowest. Perceived social support was also moderate (4.65–4.82) across all domains, highest for family and significant others. Resilience and perceived social support were positively correlated (r = 0.616, *p* < 0.001), with Hardiness most strongly associated with overall resilience (r = 0.899). Support from a significant other was particularly linked to adaptability (r = 0.617). Participants living in urban areas and those with higher income reported significantly higher resilience and social support, though with low effect sizes. No other sociodemographic associations were observed. **Conclusions**: Breast cancer survivors in this Serbian cohort reported moderate resilience and social support, with a strong interrelationship between the two. These findings underscore the importance of strengthening social support networks as a potential pathway to enhance resilience and psychological well-being in cancer survivorship care.

## 1. Introduction

Breast cancer is the most common malignancy among women, and advances in early detection and treatment have led to a growing population of survivors [1]. The aftermath of cancer often entails substantial psychosocial stress, as survivors frequently experience long-term effects—including chronic fatigue, pain, cognitive and sleep disturbances, sexual dysfunction, depression, anxiety, and persistent fear of recurrence—which can significantly undermine their quality of life and overall well-being [1,2,3]. In Eastern European settings, where survivorship resources may be limited, addressing these psychological needs is especially important.

Resilience and social support are recognized as key protective resources that help survivors adapt and recover [1]. Resilience—broadly defined as an individual’s capacity to maintain or regain psychological well-being in the face of adversity—and perceived social support (the sense of being cared for by family, friends, and others) have both been linked to better outcomes in cancer [1,4]. For example, higher resilience and robust support networks have been associated with improved general health and functioning in cancer patients [4,5]. One recent study found that greater resilience scores corresponded to fewer symptoms and higher quality of life, while satisfaction with social support from partners and family improved coping with the disease [4,5]. In short, resilient patients with strong social support tend to experience less distress and faster psychosocial recovery after breast cancer treatment.

Two well-established instruments have been proposed in the literature that measure social support and resilience among cancer patient survivors. The Connor–Davidson Resilience Scale (CD-RISC-25) is a 25-item self-report questionnaire (5-point Likert scale) in which higher scores indicate greater resilience [6]. It has demonstrated strong psychometric properties in diverse samples. For example, the CD-RISC shows high internal consistency and reliably distinguishes individuals with higher versus lower resilience [7]. The Multidimensional Scale of Perceived Social Support (MSPSS) is a 12-item inventory assessing perceived support from three sources (family, friends, and significant others) [8]. The MSPSS is widely used internationally and has consistently shown good validity and reliability across cultural settings [8]. These tools allow quantification of resilience and support levels in a standardized way. Despite the widespread use of the CD-RISC and MSPSS, validation studies in Eastern European cancer survivor populations are scarce. Cultural and socio-economic factors can affect how individuals perceive and report social support and resilience, so instruments developed elsewhere may not directly translate.

To our knowledge, no previous research has evaluated the psychometric properties using the CD-RISC-25 or MSPSS in Serbian breast cancer survivors. We conducted a cross-sectional analysis of female breast cancer survivors treated at the General Hospital Sombor. Specifically, the study aims to assess the psychometric validity of the CD-RISC-25 and MSPSS instruments within a cohort of breast cancer survivors in Serbia; quantify levels of psychological resilience and perceived social support among participants; and investigate the associations between these constructs and key sociodemographic variables, including place of residence and income. Also, this cross-sectional study aimed to investigate the relationship between resilience and perceived social support in women who have survived breast cancer. By addressing these objectives, the study seeks to enhance understanding of resilience and support mechanisms in this population and inform the development of culturally tailored psychosocial interventions.

## 2. Materials and Methods

Participants were recruited from the oncology outpatient services at General Hospital Sombor between January 2022 and December 2024. Eligible participants were adult women aged 18 years or older, who gave informed consent, participants of NALOR (National association for those treated with cancer) with a histologically confirmed diagnosis of breast cancer who had completed all primary treatments—including surgery, chemotherapy, and/or radiotherapy—at least six months prior to enrollment. Participants had to be in clinical remission at the time of the study and possess adequate cognitive and language skills to understand and complete the questionnaires in Serbian. Women with a current recurrence of breast cancer, known severe psychiatric conditions, or significant cognitive impairments were excluded from the study. A total of 265 patients were screened for eligibility. Of these, 72 were excluded for not meeting the inclusion criterion of being at least six months into post-primary treatment, resulting in a final sample of 193 participants. The study was approved by the Ethics Committee of the Medical faculty of Novi Sad and General Hospital Sombor (01-39/212/1).

### 2.1. Data Collection

Data were collected using consecutive sampling with a nearly 80% response rate. Participants were recruited during routine control examinations and, after providing informed consent, completed a structured sociodemographic and clinical questionnaire (including age, education level, employment status, marital status, place of residence, cancer stage at diagnosis, type of treatment received, and time since treatment completion), the CD-RISC-25, and the MSPSS. The survey was administered via a secure online platform on tablets provided at the clinic. Participation was voluntary and anonymous, as no identifying information or IP addresses were collected.

Resilience was assessed using the CD-RISC-25, a validated 25-item self-report measure that evaluates various components of resilience, including personal competence, tolerance to negative affect, positive acceptance of change, control, and spiritual influences. Each item is rated on a 5-point Likert scale (0–4), yielding a total score ranging from 0 to 100, with higher scores indicating greater resilience [9].

Perceived social support was measured using the MSPSS, which consists of 12 items divided into three subscales: support from family, friends, and significant others. Responses are scored on a 7-point Likert scale (1–7), with higher scores indicating higher levels of perceived social support [10].

These instruments were translated and culturally adapted following standard forward-backward translation procedures. Although previously validated Serbian versions exist in the literature, we also assessed the internal consistency reliability of both scales in our sample, calculating Cronbach’s alpha coefficients to confirm their suitability.

### 2.2. Statistical Analysis

Statistical analysis was conducted using SPSS^®^ 26.0 software (SPSS Inc., Chicago, IL, USA). Descriptive statistics were employed to calculate absolute frequencies, corresponding percentages, mean values (M), and standard deviations (SD), based on the nature of the variables. The normality of all continuous variables was tested using the Shapiro–Wilk test. To assess differences, the Mann–Whitney U test was used for comparing mean values of ordinal variables, while the Chi-square test was applied to evaluate differences between categorical variables. A significance level of *p* < 0.05 was considered statistically significant. Pearson correlation coefficients were employed to examine relationships between variables. All statistical analyses were adjusted using the Bonferroni correction to account for multiple comparisons, and the study data were corrected accordingly. Prior to analysis, the normality of the relevant data distributions was assessed using the Shapiro–Wilk test and inspection of Q-Q plots. Variables meeting normality assumptions were analyzed using Pearson correlation; otherwise, non-parametric alternatives were considered. Data processing and presentation were carried out using Microsoft Office 2021 software.

## 3. Results

### 3.1. Validity Analysis of the Scales

The validity analysis of the Connor–Davidson Resilience Scale (CD-RISC-25) was conducted by calculating the Cronbach’s Alpha coefficient. The results of the analysis are presented in Table 1.

Based on the validity analysis, we can conclude that the questionnaire is valid and suitable for assessing resilience. The highest coefficient value (α = 0.943) was obtained for overall resilience (CD-RISC-25), indicating exceptional internal consistency of the items measuring this construct. Among the subscales, the lowest coefficient value was recorded for the “Coping” subscale (α = 0.770), while the highest was noted for the “Resilience” subscale (α = 0.889).

The validity analysis of the Multidimensional Scale of Perceived Social Support (MSPSS) was also conducted. The results of the analysis are presented in Table 2.

Based on the validity analysis, we can conclude that the questionnaire is valid and suitable for assessing perceived social support. The highest coefficient value (α = 0.955) was obtained for the overall perceived social support (MSPSS), also indicating exceptional internal consistency of the items measuring this construct. Among the subscales, the lowest coefficient value was recorded for the “Significant other” subscale (α = 0.917), while the highest was noted for the “Friends” subscale (α = 0.923).

### 3.2. Levels of Resilience and Perceived Social Support

Table 3 illustrates the levels of perceived social support and resilience within the sample (N = 193). The mean values for overall perceived social support, and its domains ranged between 4.65 and 4.82 which indicates that participants perceived a moderate overall social support, and across all three domains (Significant Other, Family, and Friends). The analysis of standard deviation values, which ranged between 1.43 and 1.60, also indicated a low variability of overall, and perceived social support across all three dimensions. In terms of resilience (CD-RISC-25), participants demonstrated a moderate overall resilience score (Mean ± SD = 57 ± 18), with “Coping” (Mean ± SD = 12.19 ± 3.83) emerging as the strongest dimension. In contrast, “Optimism” (Mean ± SD = 4.02 ± 1.97) was the lowest. The mean score of overall resilience is below the general U.S. population’s average of 79 suggesting that participants have a generally lower level of resilience compared to the U.S. population.

### 3.3. Correlation Analysis of Scale Items

Based on the results, it can be observed that the overall resilience (CD-RISC-25) significantly (*p* < 0.001) and positively correlates with various dimensions of resilience, exhibiting high correlation coefficients (r > 0.5). (Table 4) The “Hardiness” domain showed the strongest correlation with the overall resilience (CD-RISC-25) (r = 0.899), identifying it as the most significant factor contributing to the overall resilience level among the participants. Additionally, “Hardiness” and “Purpose” domains demonstrated the strongest correlation among the domains (r = 0.731), indicating that within our sample, a strong sense of purpose in life was strongly linked to an individual’s level of hardiness. In contrast, “Optimism” and “Regulation of Emotion and Control” exhibited a moderate (r = 0.331) correlation; however, the weakest correlation among dimensions implying that while there was a correlation between optimism and resilience, it was relatively weak compared to other domains.

Based on the correlation results (Table 5), it can be observed that overall social support significantly (*p* < 0.001) and positively correlated with the dimensions of social support, showing high correlation coefficients (r > 0.5). The “Significant Other” dimension exhibited strongest correlation with the overall MSPSS scale (r = 0.922), indicating that the perception of support from a significant other contributed the most to the overall perception of social support among participants. The dimensions of “Family” and “Significant Other” showed the strongest correlation between them (r = 0.774), suggesting a strong connection between the support perceived from family and from a “significant other”. “Family” and “Friends” domains, however, exhibited the weakest (r = 0.729), but still a high correlation.

The Table 6 presents the correlation coefficients between the items of the two scales—CD-RISC-25 (Resilience Scale) and the MSPSS (Multidimensional Scale of Perceived Social Support)—as well as their respective domains. Based on the results, a statistically significant correlation (*p* < 0.001) was found between overall resilience and perceived social support. The correlation is positive and of a high level (r = 0.616), indicating that a greater perception of social support was generally accompanied by higher levels of resilience among our participants. Support from a “significant other” exhibited the strongest association with the “Adaptability” domain (r = 0.617), suggesting that the perception of support from a “significant other” is closely linked to the ability to adapt and cope with challenges.

### 3.4. Correlation Analysis Between the Sociodemographic Factors, Resilience and Perceived Social Support

Based on the results of the analysis (Table 7), the only statistically significant correlation (*p* < 0.05) was found between the “Place of Residence” and the “Significant Other” domain of the perceived social support. The correlation is positive, but low in level (ρ < 0.3) portraying a minor connection which suggests that, within our sample, participants who live in the city also reported a higher perceived social support originating from their life partners.

Based on the results (Table 8) of the analysis, no statistically significant correlations (*p* > 0.05) were found between the sociodemographic variables and overall, as well as specific domains of resilience measured by CD-RISC-25 score.

### 3.5. Differences in Perceived Social Support and Resilience in Relation to Sociodemographic Factors

Given that the normality of distribution assumption was not met, the Mann–Whitney U test was performed to compare perceived social support (and its domains) and resilience with sociodemographic variables (Table 9). The analysis revealed statistically significant differences only for the place of residence, and level of income. Within our sample, participants who lived in the city reported higher resilience and perceived social support (overall, and across all domains) with low-level effect sizes (r = 0.164–0.239).

Similar results were obtained for the level of income, with higher-income participants reporting higher resilience, as well as overall perceived social support (and within “Significant Other” and “Friend” domains) with low-level effect sizes, however (r = 0.148–0.156).

## 4. Discussion

The results of this study demonstrate that both the CD-RISC-25 and the MSPSS exhibit robust psychometric validity and reliability for evaluating psychological resilience and perceived social support in an oncology patient population. Despite these instruments’ strong methodological properties, descriptive analyses indicated only moderate levels of resilience and perceived social support within the cohort. The mean CD-RISC-25 resilience score was lower than the normative mean of 79 observed in the general U.S. population [9]. This finding is consistent with prior literature [11,12,13] suggesting that individuals undergoing oncological treatment frequently exhibit diminished psychological resilience, likely attributable to the cumulative physical, emotional, and psychosocial stressors associated with malignancy and its management. Within the resilience subscales, the highest mean was observed in the “Coping” and “Hardiness” domains, indicating a relative preservation of adaptive behavioral responses to stress. Conversely, the “Optimism” subdomain yielded the lowest mean score, highlighting potential cognitive-affective vulnerabilities that may merit further psychosocial support interventions.

The perceived social support scores were relatively uniform across the three domains, with slightly higher support reported from friends and lower from a significant other. Although perceived support was moderate overall, the relatively low standard deviations indicate consistent perceptions across the sample, potentially reflecting the influence of shared experiences within the clinical setting or cultural norms regarding family involvement in care. Correlation analyses further reinforce the construct validity of both scales. Overall resilience was highly correlated with its subdomains, particularly “Hardiness,” which emerged as the most influential factor. This suggests that characteristics such as perseverance and self-efficacy are central to psychological resilience in this context [9,11,12,13]. Similarly, perceived social support was strongly associated with its individual components, with support from a significant other demonstrating the strongest association with the total MSPSS score. This highlights the importance of close emotional relationships during periods of medical adversity. Importantly, a significant positive correlation between overall resilience and perceived social support was observed (r = 0.616, *p* < 0.001), indicating that patients who perceive greater social support also tend to demonstrate higher resilience. This finding is consistent with a growing body of literature [1,2,3,4,5,6,7]. suggesting that social support serves as a protective factor that can buffer the psychological impact of illness and enhance adaptive coping mechanisms. In particular, the domain of “Adaptability” within the resilience scale was most strongly associated with support from a significant other, suggesting that emotional closeness and the perception of being understood may play a key role in flexible stress response.

Sociodemographic analyses revealed that participants living in urban areas and those with higher income reported significantly higher resilience and perceived social support, though with small effect sizes (r = 0.148–0.239). No other sociodemographic variables, including treatment duration, were significantly associated with resilience or perceived social support. These findings suggest that access to resources and social networks may partially account for differences in psychosocial outcomes among oncology patients [14,15,16].

These findings underscore the complex interplay between psychological resilience, social support, and sociodemographic context in oncology patients [17]. They also highlight potential intervention points, such as fostering supportive relationships and enhancing coping skills, especially in patients who may be at greater psychosocial risk due to limited resources or prolonged treatment courses [18]. Future research should consider longitudinal designs to better understand causal relationships and changes over time, as well as qualitative studies to explore the subjective experiences underlying these quantitative associations.

This study has several limitations. Its cross-sectional design precludes any conclusions about causality between resilience and perceived social support. Furthermore, a large number of bivariate correlations were conducted without adjustment for multiple comparisons, which increases the risk of Type I error and the possibility of spurious findings. The sample was recruited from a single clinical center, potentially limiting the generalizability of the results to broader populations. Additionally, self-report measures may be subject to social desirability and recall biases. Data were collected using an online survey platform, which may have influenced participants’ responses. No patients over 70 years of age were included, although breast cancer incidence increases with age. Additionally, participants were recruited only from urban and suburban areas, limiting the generalizability of the findings to rural populations. Despite these limitations, the study has notable strengths. It represents one of the first efforts to systematically examine the relationship between resilience and perceived social support among patients in Serbia providing novel insights within this context. The use of validated instruments and a clinically relevant population enhances the internal validity of the findings. Future research should build upon these results by including multi-center and longitudinal designs to explore causal pathways and confirm generalizability across different settings.

## 5. Conclusions

The CD-RISC-25 and MSPSS are reliable tools for assessing resilience and social support in oncology patients. Despite this, participants showed only moderate levels of both, with resilience notably lower than population norms—especially in optimism, indicating emotional vulnerability. A strong correlation between resilience and social support highlights the protective role of close relationships. Sociodemographic factors like urban residence, higher income, and longer treatment were modestly linked to better outcomes. Clinically, routine psychosocial screening using these tools could help identify patients at risk of low resilience and social support, guiding early referral to tailored interventions aimed at improving psychological well-being and survivorship outcomes.

## Figures and Tables

**Table 1:** Validity Analysis of Connor–Davidson’s Resilience Scale.

Scale	Items	α
CD-RISC-25	25	0.943
Adaptability	3	0.800
Coping	5	0.770
Purpose	4	0.811
Hardiness	7	0.889
Optimism	2	0.815
Regulation of Emotion and Behaviour	2	0.802
Self-Efficacy	2	0.804

**Table 2:** Validity Analysis of Multidimensional Scale of Perceived Social Support.

Scale	Items	α
MSPSS	12	0.955
Significant other	4	0.917
Family	4	0.918
Friends	4	0.923

**Table 3:** Levels of Resilience and Perceived Social Support within the sample (N = 193).

Scales	Mean ± SD	Min–Max
**MSPSS**	4.75 ± 1.43	1–7
Significant Other	4.77 ± 1.60	1–7
Family	4.82 ± 1.52	1–7
Friends	4.65 ± 1.59	1–7
**CD-RISC-25**	57 ± 18	16–95
Adaptability	6.38 ± 2.81	1–11
Coping	12.19 ± 3.83	3–20
Purpose	9.10 ± 3.67	0–16
Hardiness	15.93 ± 5.92	2–27
Optimism	4.02 ± 1.97	0–7
Regulation of Emotion and Control	4.74 ± 1.85	1–8
Self-Efficacy	4.75 ± 2.07	0–8

**Table 4:** Pearson’s Correlation analysis of CD-RISC-25.

	CD-RISC-25	A	C	P	H	O	REC	S-E
CD-RISC-25	1							
A	0.810 **	1						
C	0.815 **	0.653 **	1					
P	0.848 **	0.622 **	0.600 **	1				
H	0.899 **	0.663 **	0.608 **	0.731 **	1			
O	0.589 **	0.456 **	0.473 **	0.436 **	0.413 **	1		
REC	0.698 **	0.481 **	0.546 **	0.543 *	0.572 **	0.331 **	1	
S-E	0.787 **	0.577 **	0.574 **	0.614 **	0.683 **	0.433 **	0.588 **	1

** Correlation is significant at the ≤0.01 level (2-tailed) after Bonferroni’s correction. * Correlation is significant at the ≤0.05 level (2-tailed) after Bonferroni’s correction. CD-RISC-25—Overall resilience; A—Adaptability; C—Coping; P—Purpose; H—Hardiness; O—Optimism; REC—Regulation of Emotion and Control; S-E—Self-Efficacy.

**Table 5:** Pearson’s Correlation Analysis of Multidimensional Scale of Perceived Support.

	MSPSS	SO	Fa	Fr
MSPSS	1			
SO	0.922 **	1		
Fa	0.911 **	0.774 *	1	
Fr	0.905 **	0.747 **	0.729 **	1

** Correlation is significant at the ≤0.01 level (2-tailed) after Bonferroni’s correction. * Correlation is significant at the ≤0.05 level (2-tailed) after Bonferroni’s correction. MSPSS—Overall perceived social support; SO—Significant Other; Fa—Family; Fr—Friends.

**Table 6:** Pearson’s Correlation between the Resilience and Perceived Social Support.

	CD-RISC-25	A	C	P	H	O	REC	S-E
MSPSS	0.616 **	0.581 **	0.529 **	0.490 **	0.512 **	0.369 **	0.399 **	0.541 **
SO	0.617 **	0.576 **	0.532 *	0.503 **	0.516 **	0.350 **	0.376 **	0.561 **
Fa	0.542 **	0.510 **	0.412 **	0.421 **	0.469 *	0.380 **	0.370 **	0.475 **
Fr	0.525 **	0.503 **	0.502 **	0.415 **	0.416 **	0.283 **	0.347 **	0.442 **

** Correlation is significant at the ≤0.01 level (2-tailed) after Bonferroni’s correction. * Correlation is significant at the ≤0.05 level (2-tailed) after Bonferroni’s correction. MSPSS—Overall perceived social support; SO—Significant Other; Fa—Family; Fr—Friends; CD-RISC-25—Overall resilience; A—Adaptability; C—Coping; P—Purpose; H—Hardiness; O—Optimism; REC—Regulation of Emotion and Control; S-E—Self-Efficacy.

**Table 7:** Spearman’s Correlation between the Sociodemographic factors and Perceived Social Support (N = 193).

	A	EL	ES	PR	IL	RS	L	TSCT	CR
MSPSS	−0.024	0.035	0.045	0.210	0.155	0.048	−0.102	0.031	−0.041
SO	−0.005	0.031	0.025	0.231 *	0.151	0.021	−0.071	−0.008	−0.037
Fa	−0.019	0.019	0.010	0.195	0.118	0.105	−0.131	0.017	−0.052
Fr	−0.025	0.022	0.078	0.163	0.145	−0.005	−0.069	0.066	0.018

* Correlation is significant at the ≤0.05 level (2-tailed) after Bonferroni’s correction. MSPSS—Overall perceived social support; SO—Significant Other; Fa—Family; Fr—Friends; A—Age; EL—Education level; ES—Employment status; PR—Place of Residence; LI—Level of Income; RS—Relationship Status; L—Lifestyle; TSCT—Time Since Cancer Treatment; CR—Cancer Recurrence.

**Table 8:** Spearman’s Correlation between the Sociodemographic factors and Resilience (N = 193).

	A	EL	ES	PR	IL	RS	L	TSCT	CR
CD-RISC-25	−0.060	−0.034	0.050	0.195	0.157	0.056	−0.135	−0.019	−0.101
A	−0.039	0.039	−0.022	0.177	0.194	0.046	−0.138	0.060	−0.064
C	−0.052	0.009	0.084	0.185	0.086	0.031	−0.032	0.014	−0.047
P	−0.028	−0.054	0.087	0.180	0.139	0.040	−0.097	−0.005	−0.107
H	−0.079	−0.061	0.015	0.175	0.202	0.065	−0.129	−0.078	−0.107
O	−0.084	−0.114	0.029	0.173	0.016	0.130	−0.163	0.039	−0.057
REC	0.087	−0.041	−0.010	0.140	0.082	−0.027	−0.076	0.002	−0.057
S-E	−0.029	−0.030	−0.007	0.080	0.088	0.017	−0.119	−0.016	−0.035

A—Age; EL—Education level; ES—Employment status; PR—Place of Residence; LI—Level of Income; RS—Relationship Status; L—Lifestyle; TSCT—Time Since Cancer Treatment; CR—Cancer Recurrence; CD-RISC-25—Overall resilience; A—Adaptability; C—Coping; P—Purpose; H—Hardiness; O—Optimism; REC—Regulation of Emotion and Control; S-E—Self-Efficacy.

**Table 9:** Effects of Sociodemographic factors on Perceived Social Support, and Resilience (N = 193).

Sociodemographic Factors			MSPSS	Significant Other	Family	Friends	CD-RISC-25
		N	Mdn ± IQR				
Age	41–50	75	5 (2)	5 (2.5)	5 (2.5)	4.62 (1.67)	59 (26)
	51–60	50	4.75 (2.5)	5 (3)	5.25 (1.5)	4.82 (1.62)	59 (32)
	61–70	68	4.75 (2)	5 (2.5)	5 (2.75)	4.55 (1.47)	55 (28)
	*p* values		0.689	0.989	0.386	0.663	0.346
Education Level	Secondary	106	4.50 (2)	5.00 (2.5)	5 (2.5)	4.50 (3)	58 (27)
	Highschool/College	87	5.00 (2)	5.00 (3)	5 (2.5)	5 (2.5)	58 (25)
	*p* values		0.676	0.528	0.663	0.568	0.639
Employment Status	Unemployed	102	4.50 (2)	5 (2.5)	5 (3)	5 (3)	54 (28)
	Employed	91	5 (2)	5 (2)	5 (2)	5 (3)	59 (27)
	*p* value		0.490	0.685	0.880	0.298	0.489
Place of Residence	Town	55	4 (1.5)	4 (2.5)	4.50 (2)	4.50 (2.5)	52 (26)
	City	138	5 (3)	5 (3)	5 (2.5)	5 (3.5)	61 (28)
	*p* values		0.003	<0.001	0.012	0.022	0.007
Level of Income	Below average	61	4 (3)	4.50 (2.5)	4.50 (2.5)	4.50 (3)	53 (23)
	Above average	132	5 (2.5)	5 (2.75)	5 (2.5)	5 (2.5)	59 (27)
	*p* value		0.034	0.039	0.111	0.032	0.030
Relationship Status	Single	52	4.75 (2)	5 (2.5)	4.75 (3)	5 (2.5)	56 (29)
	Married	141	5 (2)	5 (3)	5 (2)	4.50 (3)	58 (26)
	*p* value		0.508	0.774	0.145	0.941	0.442
Lifestyle	With partner	120	5 (2)	5 (3)	5 (2)	5 (3)	61 (27)
	Other	73	4.50 (2)	4.75 (2.75)	4.50 (2.5)	5 (2.5)	53 (28)
	*p* value		0.161	0.328	0.070	0.337	0.061
TSCT	Less than 3 years	103	4.50 (2)	5 (3)	5 (2)	4.50 (3)	59 (27)
	More than 3 years	90	5 (2)	4.75 (2.5)	5 (3)	5 (2)	56 (26)
	*p* value		0.663	0.911	0.817	0.361	0.790
CR	Yes	52	5 (2)	5 (2.5)	5 (2)	5 (3)	58 (27)
	No	141	4.50 (2)	4.50 (3)	4.50 (2.5)	5 (2.5)	54 (30)
	*p* value		0.573	0.613	0.475	0.804	0.161

A—Age; EL—Education level; ES—Employment status; PR—Place of Residence; LI—Level of Income; RS—Relationship Status; L—Lifestyle; TSCT—Time Since Cancer Treatment; CR—Cancer Recurrence; CD-RISC-25—Overall resilience; MSPSS—Overall perceived social support; SO—Significant Other; Fa—Family; Fr—Friends.

## Data Availability

The data presented in this study are available on request from the corresponding author. The data are not publicly available due to privacy and ethical considerations.
